# Microbial metabolism of dietary components to bioactive metabolites: opportunities for new therapeutic interventions

**DOI:** 10.1186/s13073-016-0296-x

**Published:** 2016-04-21

**Authors:** Linda S. Zhang, Sean S. Davies

**Affiliations:** Division of Clinical Pharmacology, Vanderbilt University, Nashville, TN 37232 USA; Department of Pharmacology, Vanderbilt University, Nashville, TN 37232 USA; Vanderbilt Institute of Chemical Biology, Vanderbilt University, Nashville, TN 37232 USA

## Abstract

Mass spectrometry- and nuclear magnetic resonance-based metabolomic studies comparing diseased versus healthy individuals have shown that microbial metabolites are often the compounds most markedly altered in the disease state. Recent studies suggest that several of these metabolites that derive from microbial transformation of dietary components have significant effects on physiological processes such as gut and immune homeostasis, energy metabolism, vascular function, and neurological behavior. Here, we review several of the most intriguing diet-dependent metabolites that may impact host physiology and may therefore be appropriate targets for therapeutic interventions, such as short-chain fatty acids, trimethylamine *N*-oxide, tryptophan and tyrosine derivatives, and oxidized fatty acids. Such interventions will require modulating either bacterial species or the bacterial biosynthetic enzymes required to produce these metabolites, so we briefly describe the current understanding of the bacterial and enzymatic pathways involved in their biosynthesis and summarize their molecular mechanisms of action. We then discuss in more detail the impact of these metabolites on health and disease, and review current strategies to modulate levels of these metabolites to promote human health. We also suggest future studies that are needed to realize the full therapeutic potential of targeting the gut microbiota.

## Alterations in microbial transformation of dietary components associate with disease

The symbiotic relationship between mammals and the trillions of microbial cells that reside in their gastrointestinal tracts relies on a complex molecular dialogue, with microbial metabolites acting as major mediators of this dialogue. Essential roles for several microbial metabolic pathways in host physiology have been long established, including in the production of vitamin K, the production of water-soluble B vitamins including biotin, folates, nicotinic acid, pyridoxine, riboflavin, cobalamin and panthotenic acid, the degradation of dietary oxalates, and modification of bile salts (reviewed in [1, 2]). However, intense interest in the gut microbiota over the past decade has led to the discovery of many new areas where bacterial transformation of dietary components may play critical roles in host health and disease. This increased understanding of diet–microbiota–host interactions suggests significant opportunities to create new therapeutic approaches, including selectively altering the microbial production of molecules to promote human health and prevent disease [3].

Elucidating target microbial metabolites that modulate host physiology requires identifying the major metabolites (and their downstream co-metabolites formed by the phase I/II xenobiotic metabolizing enzymes of their host) that differ between healthy and diseased individuals, and assessing the biological activities of these metabolites. A series of landmark metabolomics studies over the past decade have significantly advanced our understanding by using mass spectrometry (MS) or nuclear magnetic resonance (NMR) analysis to identify potentially important microbial metabolites that derive from the gut microbes [4–10], that are enriched or depleted in diseased individuals [11–30], or that can be used to predict physiological response to foods or other interventions [31, 32] (Table 1). These studies have identified a number of metabolites that may play important roles in human health and disease, including short-chain fatty acids (SCFAs) and long-chain fatty acid metabolites such as conjugated linoleic acid (CLA) and 10-hydroxy-*cis*-12-octadecenoate (HYA); trimethylamine (TMA) and trimethylamine *N*-oxide (TMAO); tryptophan metabolites such as indole, indole-3-propionate (IPA) and indoxyl-sulfate (IndsS); and tyrosine and phenylalanine metabolites such as hippuric acid, phenylacetylglycine, phenyl sulfate, para-cresyl sulfate (PCS), phenylpropionylglycine, cinnamoylglycine and equol sulfate. Many of the metabolites identified by these studies result from the transformation of specific dietary components by select species of microbes that express the necessary enzymes to act on these components. Thus, the variable presence of microbes utilizing these diet-dependent metabolic pathways may be key to understanding the variable host response to specific dietary components and susceptibility to disease [32].

**Table 1:** Key microbial metabolomic studies of the past decade

Studies identifying major gut microbial metabolites				
Aim of study		Population	Results	Reference
Identify metabolites modulated by gut microbiota in various tissue and fluids		Conventional versus germ-free C3H/HeJ mice	•Higher bile acid levels in gut of germ-free mice. •Higher phosphocholine and glycine in liver of germ-free mice. •Hippurate and 5-aminovalerate reduced in germ-free animals. •Higher levels of betaine, choline and myo-inositol in kidney of germ-free mice.	[4]
Determine effect of antibiotic treatment on metabolome		Normal versus vancomycin-treated female NMRI mice	•Vancomycin reduced urinary levels of hippurate, phenylacetylglycine, taurine, TMA and TMAO, and increased urinary levels of α-ketoisovalerate, *n*-butyrate, creatinine, guanidoacetic acid and glycine. •Vancomycin reduced fecal levels of uracil, amino acids, SCFAs and urinary phenylacetylglycine and hippurate.	[5]
Identify metabolites derived from the gut microbiota		Conventional versus germ-free Swiss Webster mice	•Metabolites highly enriched or only present in conventional mice include indole derivatives (such as indoxyl sulfate and IPA), phenyl/benzoate derivatives. (hippurate, p-cresol), and conjugated fatty acids.	[6]
Identify serum metabolites derived from gut microbiota		Conventional versus germ-free Swiss Webster mice	•Increased serum metabolites related to energy metabolism (pyruvic acid, citric acid, fumaric acid, malic acid) in conventional compared to germ-free mice.	[7]
Determine effect of antibiotics on metabolite production		Normal versus penicillin- and streptomycin-treated Han–Wistar rats	•Antibiotics reduced urinary excretions of hippurate, phenylpropionic acid, phenylacetylglycine and indoxyl-sulfate, and elevated urinary excretions of taurine, glycine, citrate, 2-oxoglutarate and fumarate. •Antibiotics reduced fecal SCFA.	[8]
Identify fecal and urinary metabolites derived from the gut microbiota		Normal versus imipenem/cilastatin Wistar rats	•Antibiotic treatment altered 202 urinary and 223 fecal metabolites. •Major classes reduced by antibiotics include SCFAs, phenyl/benzoates (for example, p-hydroxyphenylacetate, m-hydroxyphenylacetate, hydroxycinnamic acid, phynylvalerate, p-aminobenzoate and hippurate), and indole-containing substances (indoxyl sulfate, indole-acetate, indole-carboxylate and indole-acetaldehyde), and urobilin. Antibiotic treatment increased tryptophan and tryptamine in feces.	[9]
Compare metabolomes of human versus humanized and conventionally raised mice.		Germ-free versus humanized versus conventional Swiss Webstermice	•Metabolome of humanized mice was more similar to metabolome of human donors than to metabolome of conventional mice, with more differences in feces than urine. •Humanized mice had higher fecal levels of tryptamine and indoxyl glucuronide, and lower levels of trisaccharide, creatine and creatinine than conventional mice.	[10]
Studies examining microbial metabolites enriched or depleted in disease states				
Disease	Aim of study	Population	Results	Reference
Metabolic disorders and CVD	Identify metabolites associated with fatty liver disease	Disease-susceptible (129S6) versus disease-resistant (BALBc) mice	•Increased urinary dimethylamine, TMA, TMAO, formate and hippurate in 129S6 mice on HFD. •Decreased plasma phosphatidylcholine seen in 129S6 mice likely due to microbial conversion to TMA.	[11]
	Identify metabolites associated with obesity	Fecal transplantation from ob/ob, ob/+, +/+ C57BL/J mice to germ-free mice	•Recipients of fecal transplant reciprocate phenotype of donor. Cecal levels of acetate and butyrate increased in obese mice.	[12]
	Identify urinary metabolites associated with obesity	Lean versus obese Zucker rat	•Obese mice have higher urinary creatinine, TMAO, hippurate and acetate.	[13]
	Identify metabolites associated with obesity	Healthy versus obese insulin-resistant male humans	•Increased microbiota-derived hippurate acid, trigonelline, 2-hydroxyisobutyrate and xanthine was seen in the obese microbiota	[14]
	Identify metabolites that predict CVD	Human subjects with CVD	•Three metabolites of dietary phosphatidylcholine (choline, TMAO, betaine) predict risk for CVD. •Studies in mice confirmed critical role for dietary choline and gut flora in TMAO production and CVD.	[15]
	Identify metabolites elevated in mice highly susceptible to diet-induced obesity.	C57J versus C57N mice	•C57N more susceptible to diet-induced obesity than C57J. •In cecum, C57N have decreased taurine-conjugated bile acids, bile acid sulfates, enterolactone and enterodiol; altered arachidonate metabolites and increased urobilins. •In liver, C57N have increased taurine-conjugated bile acids, fatty acids and urobilins.	[16]
	Identify urinary metabolite associated with human adiposity	Human subjects from INTERMAP study (*n* = 2324)	•Urinary metabolites associated with increased BMI included *N*-acetyl neuraminate, TMA, PCS, succinate, citrate ethanolamine.	[17]
	Determine effects of bariatric surgery on metabolome	Severely obese human subjects undergoing bariatric surgery	•Bariatric surgery reversed most metabolites associated with obesity such as increased aromatic and branched-chain amino acids, pyruvate, citrate, formate, methanol and isopropanol.	[18]
	Determine effect of prebiotics in maternal diet on offspring adiposity	Female Sprague Dawley rats fed high-fat/sucrose diet with and without 10% oligofructose	•Addition of 10% oligofructose to diet normalizes body weight in diet-induced obese dams and inhibited adiposity in offspring. •Microbiota composition of offspring similar to dams. •Diet-induced obese dams have increased SCFAs, glycine, betaine, 2- and 3-hydroxybutyrate, cytidine, o-phosphocholine, formate, acetone and reduced levels or carnitine, methanol, amino acid, lactate and *O*-phosphorylcholine •Subsequent addition of 10% oligofructose reduced *O*-phosphorylcholine, acetone, cytidine and 3-hydroxybutyrate, and increased propionate, urea, myo-inositol, isobutyrate, alanine, methionine, ornithine and proline.	[19]
Inflammatory bowel disorders	Identify metabolites associated with Crohn’s disease	Human twin pairs	•In feces, twins with Crohn’s disease have increased fecal levels of hydroxyphenylacetylglycine, tyrosine, tryptophan, glycocholate, fatty acids and phenylalanine metabolites	[20]
	Identify metabolites associated with IBS	Human subjects with IBS versus healthy	•In feces, individuals with IBS have increased bile acid and decreased branched-chain fatty acids. Trends of increased taurine and cadaverine in ulcerative colitis. •No change detected in SCFAs and amino acids.	[21]
	Identify metabolites specific to Crohn's disease, ulcerative colitis or pouchitis	Diseased versus healthy human subjects	•Medium-chain fatty acids and some protein fermentation metabolites decreased in Crohn’s disease, ulcerative colitis and pouchitis. •Hexanoate inversely correlated with Crohn’s disease. •Styrene positively correlated with ulcerative colitis.	[22]
	Develop simplified metabolomics approach to discriminate ulcerative colitis from Crohn’s disease	Human	•A single analytical platform based on reverse phase UHPLG-Orbitrap HRMS provided sufficient coverage to discriminate between ulcerative colitis and Crohn’s disease in fecal samples.	[30]
CKD	Determine effect of resistant starch on the gut metabolome in CKD	Sprague Dawley rat with adenine-induced CKD fed high-fiber versus no additional fiber	•High-fiber-resistant starch diet improved kidney function and ameliorated CKD. •High-fiber-resistant starch decreased urinary indoxyl sulfate and p-cresol.	[23]
*C. difficile* infections	To determine effect of antibiotics on *C. difficile* infection	C57BL/6 mice infected with *C. difficile* given antibiotics versus no antibiotics	•Antibiotics decrease secondary bile acids, glucose, free fatty acids and dipeptides while primary bile acids and sugars increase. •Concluded that *C. difficile* exploits metabolites such as taurocholate or carbon sources for germination and growth.	[24]
	To determine how bile acids impact *C. difficile* dynamics	C57BL/6 mice infected *with C. difficile* given antibiotics versus no antibiotics	•Susceptibility to *C. difficile* occurred only after antibiotic treatment, and was accompanied by a loss of secondary bile acids. •Physiological concentrations of secondary bile acids inhibited *C. difficile* spore germination and growth.	[25]
	To analyze fecal metabolome in *C. difficile* infection	Human subjects with *C. difficile* versus healthy given antibiotics	•In feces, subjects with *C. difficile* have decreased fecal cholesterol and increased fecal coprostanol. •*63 microbes associated with increased coprostanol levels identified.*	[26]
Neurological or behavior disorders	To identify a pattern of metabolic perturbance in ASD	Children with ASD versus healthy controls	•82 metabolites were altered between ASD and controls. •In ASD children, levels of amino acids (glycine, serine, threonine, alanine, histidine, taurine) and antioxidants such as carnosine were lower.	[27]
	Determine if microbiota play a role in development of ASD	Maternal immune activation model of ASD	•Maternal immune activation treatment altered 8 % of all serum metabolites detected, with EPS most increased. •Administration of *B. fragilis* normalized behavior and EPS levels.	[28]
	Determine effects of antibiotics on cognition	C57BL/6N mice given antibiotics versus no antibiotics	•Antibiotic treatment impaired novel object recognition, but not spatial learning and memory. •Antibiotic treatment reduced colon levels of SCFAs, TMA, adenine and uracil. •Antibiotic treatment increased plasma levels of corticosterone and phospholipids, and reduced plasma levels of lysophospholipid and p-cresyl sulfate, TMAO, deoxycholate and chenodeoxycholate. •Antibiotic treatment altered brain-derived neurotrophic factor, NMDA receptor subunit 2B, serotonin transporter and neuropeptide Y system.	[29]
Studies using metabolites as predictive biomarkers of physiological response to intervention				
Aim of study		Population	Results	Reference
To create a computational platform that predicts response to dietary intervention		Obese human subjects	•The CASINO (community and systems-level interactive optimization) toolbox was able to predict and quantitatively describe altered fecal and serum SFCA and amino acid levels in response to diet intervention.	[31]
To develop a machine-learning algorithm that predicts postprandial glycemic response		Healthy human subjects	•High interpersonal variability in postprandial glycemic response •Microbial metabolites key variables in algorithm that accurately predicts personalized responses to real life meals	[32]

*ASD* autism spectrum disorder, *BMI* body mass index, *CKD* chronic kidney disease, *CVD* cardiovascular disease, *EPS* 4-ethylphenylsulfate, *HFD* high-fat diet, *IBS* irritable bowel syndrome, *IPA* indole-3-propionate, *PCS* para-cresyl sulfate, *SCFAs* short-chain fatty acids, *TMA* trimethylamine, *TMAO* trimethylamine *N*-oxide

**Table 2:** Microbial metabolites: their synthesis, mechanisms of action, and effects on health and disease

(Co-) Metabolites	Microbial phyla/species	Molecular targets	Effects on health & disease
Butyrate Synthesized predominantly via butyryl-CoA:acetate CoA transferase pathway [37]	*Bacteriodes* *Ruminococcaceae* *Lachnospiraceae*	Energy source for colonocytes Inhibits HDAC [43, 53, 54, 102] Activates GPR41 and GPR43 [38, 39] Activates GPR109A [40] Suppresses nuclear NF-kB activation [40, 46, 47] Modulates PPAR-γ [59, 102]	Increased intestinal barrier function [52, 59] Anti-inflammatory [44, 46, 62, 103] Anti-lipogenic [41] Improves insulin sensitivity [41, 102, 103] Increases energy expenditure [41, 102] Anti-cancer [51, 61]
Propionate Synthesized predominantly via succinate pathway [36]	*Propionibacterium* *Bacteroides* *Negativicutes*, *Selenomonas ruminantium,* *Roseburia inulinivorans* *Escherichia coli*	Activates GPR41 [89] and GPR43 [38, 39] Upregulates GLP-1, PYY, leptin [34] Increases oxidative stress, alters phospholipid composition, induces inflammation in the brain [179]	Anti-inflammatory [56] Anti-cancer Anti-lipogenic [41] Improves insulin sensitivity [41] Increases energy expenditure [41] Increases satiety [104] Associated with autistic spectrum disorder [179]
Acetate Synthesized directly from acetyl-CoA or from CO_2_ via the Wood-Ljungdahl pathway [34]	*Most anaerobic gut bacteria studied produce acetate*	Energy substrate Activates GPR43 [57, 58] and GPR41 [38, 39] Activates AMPK pathway [34]	Anti-inflammatory [57, 58] Anti-lipogenic [41] Improves insulin sensitivity [41] Increases energy expenditure [41] Reduces glycemia in diabetic rodent models [34] Protects against asthma [90]
TMA Cleavage from choline via CutC & CutD [108] and from *L*-carnitine via YeaW & YeaX or CntA & CntB [111]	*Desulfovibrio* *Proteus mirabilis* *Ruminococcus* *Akkermansia muciniphilia*	TAAR5 [118] Potentially others	Excessive levels lead to fish malodor syndrome
TMAO Oxidized from TMA by FMO3 in liver [120]		Osmolyte [116] Mechanisms remains unknown	Accelerates atherosclerosis [15, 112, 115] Contributes to kidney dysfunction and chronic kidney disease [116]
Indole Synthesized from tryptophan via tryptophanase	*Lactobacillus* *Bifidobacterium longum* *Bacteroides fragilis,* *Parabacteroides distasonis* *Clostridium bartlettii* *E. hallii*	Activates AhR [125] Modulates GLP-1 secretion [131]	Maintains host-microbe homeostasis at mucosal surface [125–127] Signals with intestinal L cells to influence host metabolism [131]
Indole sulfate Hepatic sulfonation from indole		Cytotoxic Produces free radicals [142] Stimulates endothelial release of microparticles [140] Enhances monocyte adhesion to vascular endothelium [141]	Induces renal and vascular dysfunction [139–141] Associated with chronic kidney disease [138] Associated with cardiovascular disease [141]
Indole-3-aldehyde Synthesized from tryptophan via unidentified enzymes	*Lactobacillus*	Activates AhR resulting in IL-22 production [125]	Maintains host-microbe homeostasis at mucosal surface [125]
IPA Synthesized from tryptophan	*Clostridium sporogenes*	Activates PXR [132] Scavenges hydroxyl radicals [134] Reduces DNA damage and lipid peroxidation in neurons [135] Inhibits beta-amyloid fibril formation [134]	Maintains intestinal barrier function and mucosal homeostasis [132] Anti-oxidant [134, 135, 137] Protects against ischemia-induced neuronal damage [134] Potential therapy for Alzheimer’s disease [134]
PCS Hepatic sulfination of p-cresol, which is synthesized from tyrosine by hydroxyphenylacete decarboxylase [144]	*Clostridium difficile*	Damages cell membranes [154] Induces apoptosis [155] Activates NADPH oxidase [156] Activates JNK and p38-MAPK [157] Activates Rho-K [158] Activate EGF receptor [159]	Accumulates in and predicts chronic kidney disease [146–149]
EPS Hepatic sulfination of 4-ethylphenol, potentially from paracoumaric acid via decarboxylase and vinyl phenol reductase or from genistein	Produced by unknown commensal bacteria	No specific molecular targets identified but assumed to be similar to para-cresol sulfate	Associated with autistic spectrum disorder [28] Potential uremic toxin [153]
HYA Derived from linoleic acid via linoleate isomerase activity [169]	*Lactobacillus plantarum*	Activates GPR40 [176] Activates Nrf2 [175]	Maintains intestinal barrier [176] Anti-inflammatory [175]
CLA CLnA Derived from linoleic acid via linoleate isomerase activity [169]	*Lachnospiraceae* *Lactobacillus* *Bifidobacteria* *Faecalibacterium prausnitzii* *Propionibacterium*	Modulates PPARγ [171] Activates PPARα [172] Inhibits cyclooxygenase and lipoxygenase [173, 174] Modulates cytokine production and T-cell responses [180]	Reduces adiposity [170] Improves insulin sensitivity [170] Anti-cancer [170] Reduces atherosclerosis [170] Anti-inflammatory [170]

*AhR* aryl hydrocarbon receptor, *AMPK* AMP kinase, *CLA* conjugated linoleic acid, *CLnA* conjugated linolenic acid, *CoA* coenzyme A, *EGF* epidermal growth factor, *EPS* 4‐ethylphenylsulfate, *GLP* glucagon-like peptide, *GPR* G-protein coupled receptor, *HDAC* histone deacetylase, *HYA* 10‐hydroxy‐cis‐12‐ octadecenoate, *IL* interleukin, *IPA* indole-3-propionate, *JNK* c-Jun N-terminal protein kinase, *MAPK* mitogen-activated protein kinase, *Nrf2* nuclear factor (erythroid-derived 2)-like 2, *PCS* para‐cresyl sulfate, *PPAR* peroxisome proliferator-activated receptor, *PXR* pregnane X receptor, *PYY* Peptide YY, *Rho-K* rho-kinase, *TMA* trimethylamine, *TMAO* trimethylamine N‐oxide

This review will focus on several key metabolites formed by the gut microbiota from dietary components that have been revealed recently to produce remarkable effects on host physiology and that are currently being targeted or have high potential to be targeted as treatments for human disease. We will describe briefly the microbial origin of these metabolites and the biological actions of these metabolites on their host. We will then discuss in more detail current and potential therapeutic approaches to manipulate these metabolite levels and broader areas of research that are needed to understand the potential value of gut microbial metabolites.

## Short-chain fatty acids

### Biosynthesis and molecular mechanisms of action

SCFAs constitute the most abundant microbial metabolite, reaching concentrations of 50–130 mM in the proximal colon [33]. The biochemical pathways leading to the formation of these SCFAs by saccharolytic microbes are reviewed in [34]. Acetate, the most abundant SCFA, is produced by many microbial species as acetyl coenzyme A (acetyl-CoA) and is central to many metabolic pathways [35]. Propionate is synthesized predominantly through the succinate pathway [36], while butyrate is synthesized predominantly via butyryl-CoA:acetate CoA transferase [37]. Because the production of SCFAs depends on complex cross-feeding of substrates and disposal of waste products such as hydrogen and carbon dioxide gas among various species of the microbial community [34], there is not a simple linear relationship between gut SCFA levels and individual dietary components or bacterial strains. This is a key point when considering therapeutic attempts to increase SCFAs, particularly because these other waste products produce significant gastrointestinal distress for the host. Thus, simply administering a single dietary component or strain of bacteria may not have the intended effect on SCFAs, and careful confirmation of alterations in SCFA levels and other products are needed to interpret the results of such studies.

A number of molecular mechanisms of action have been ascribed to acetate, propionate and butyrate that may be relevant to their therapeutic potential to promote intestinal health, reduce inflammation, and inhibit cancer (Table 2). All three SCFAs are ligands for G-protein-coupled receptor 43 (GPR43; also known as FFA2) and GPR41 (also known as FFA3), although they range in potency [38, 39]. Butyrate is also a low-affinity ligand for GPR109A (also known as hydroxycarboxylic acid receptor) [40]. These three receptors are present throughout the gastrointestinal tract, as well as on immune cells and adipose tissues, and have been implicated in the regulation of inflammation and cancer. Additionally, both propionate and butyrate inhibit histone deacetylase (HDAC) activity and thereby alter gene expression, which appears to suppress tumor formation and inflammatory pathways in many tissues. In hepatocytes and adipocytes, all three SCFAs appear to modulate peroxisome proliferator-activated receptor-γ (PPAR-γ) expression (by an unknown, indirect mechanism), which leads to increased expression of uncoupling protein-2, reduced ATP levels, and activation of AMP kinase (AMPK) [41]. Similarly, propionate modulates PPAR-γ activity in intestinal cells, one effect of which is to increase expression of epithelial Kruppel-like factor 4 [42], a tumor suppressor transcription factor that may be important in preventing colorectal cancer. Butyrate also inhibits the NF-κB pathway (a prototypical proinflammatory signaling pathway that expresses genes for cytokines, chemokines and adhesion molecules) [43–47]. All three SCFAs are used as energy substrates, with propionate serving as a substrate for gluconeogenesis while acetate and butyrate serve as substrates for fatty acid synthesis. These various actions of SCFAs allow them to exert pluripotent effects that generally promote intestinal health, reduce inflammation and inhibit cancer, and, as will be discussed below, a number of studies have investigated the therapeutic potential of SCFAs or fermentable fibers. The results of these studies have often been equivocal, suggesting that a far better understanding of the appropriate doses and the precise mechanisms by which SCFAs act in various disease states is needed to design more appropriate interventions.

### Effects on intestinal inflammation and colorectal cancer

A decrease in luminal SCFAs is associated with ulcerative colitis and intestinal inflammation, which can be ameliorated with dietary fiber or administration of SCFAs [48–50]. Reduced barrier function promotes intestinal inflammation, and butyrate promotes barrier function by inducing “physiological hypoxia” in intestinal cells via HDAC inhibition [51], which thereby stabilizes hypoxia inducible factor-1α to regulate a number of genes that improve epithelial barrier function [52]. Butyrate inhibition of HDAC also promotes intestinal immune tolerance through regulating the function of intestinal macrophages [53] and development of regulatory T cells through mechanisms that involve acetylation of forkhead box P3 (FOXP3) [54, 55] and activation of GPR43 [56]. Deletion of GPR43 exacerbates intestinal inflammation in mice [57], while GPR43 activation by acetate can also protect against colonic epithelial injury [58]. Butyrate can also modulate the expression of intestinal tight junction proteins, enhance epithelial cell proliferation, and inhibit apoptosis [59], possibly through its effects on glucagon-like peptide (GLP)-2 secretion, which is known to have a trophic effect on the epithelium [60].

Intestinal inflammation contributes to the development of colorectal cancer, and the contribution of SCFA-producing bacteria to the inhibition of colon carcinogenesis remains unresolved. Besides its anti-inflammatory effects, butyrate also exerts anti-proliferative and anti-cancer effects when tumor cell lines are exposed to it in vitro [61–63], primarily through HDAC inhibition [64, 65]. Epidemiological studies, although inconclusive, show an inverse relationship between the intake of dietary fiber and incidence of colon cancer [66–71], suggesting that increased colonic SCFAs as a result of fiber fermentation may be responsible for the protective effect. However, large randomized multicenter clinical trials, such as The Polyp Prevention Trial (*n* = 2079) [72] and the Wheat Bran Study (*n* = 1429), [73] showed no impact of a high-fiber diet on recurring polyp formation. However, other studies have shown inconsistent relationships between SCFAs and colon cancer development in humans [74–77] as well as in animals (for a critical evaluation of studies, see [78]). Butyrate has been shown to stimulate cell proliferation in a number of studies under conditions of energy deprivation [79, 80], which is likely due to butyrate being an energy source for colonic epithelial cells. Yet, under states of hyperproliferation, such as that induced by secondary bile acids [81] or in cancer cells maintained under high glucose [80], butyrate suppresses proliferation. These discrepancies can be partially explained by the fact that cancer cells predominantly use glucose rather than fatty acids such as butyrate as an energy source (the Warburg effect) [82], resulting in intracellular accumulation of butyrate that sufficiently inhibits HDAC and consequently cell growth [83]. Critical review of these conflicting and sometimes paradoxical results reached the conclusion that butyrate exerts anti-proliferative effects only at specific sensitive stages of the carcinogenesis, that these effects are dependent on delivery of butyrate to the colon, that the extent of butyrate production in the colon varies widely based on type of fiber, and that very high colonic concentrations of butyrate are required and may be difficult to reproducibly achieve in humans [78, 84]. More recent studies using mice to carefully control cancer phenotype have not resolved these issues. For instance, a mouse study used gnotobiotic mice treated with azoxymethane followed by dextran sodium sulfate (DSS) to induce colon cancer and then colonized these mice with butyrate-producing bacterium, and found that dietary fiber had a butyrate-dependent tumor-suppressive effect that required microbiota [83]. In this model, butyrate was metabolized less in tumors and functioned as a HDAC inhibitor. In contrast, another recent study showed that gut microbial production of butyrate stimulated polyp formation in a genetic mouse model of colorectal cancer (*Apc*^*Min/+*^*Msh2*^*−/−*^) [85]. Importantly, more than 10 % of colon cancers in humans carry lesions in adenomatous polyposis coli (APC) and DNA mismatch repair gene MutS homolog 2 (MSH2) [86]. Thus, whether consumption of dietary fiber to generate butyrate can be used therapeutically to prevent or treat colorectal cancer remains very much unresolved. Given the need for very high butyrate levels to be effective and the aversion most humans have for high amounts of fiber in their diets, alternative strategies such as fibers engineered to increase butyrate production with reduced hydrogen and carbon dioxide gas production may be required.

### Peripheral inflammation

The anti-inflammatory effects of SCFAs extend beyond the gut, such as inhibiting vascular smooth muscle cell proliferation and migration [87], improving kidney function [88], conferring anti-inflammatory effects in the lung [89, 90], and protecting against inflammatory arthritis [91]. The mechanisms underlying these effects appear to center largely on HDAC inhibition and GPR43 activation, and thereby act via modulating immune cell activation. For example, mice fed a high-fiber diet have increased circulating SCFAs, which protected against allergic inflammation in the lungs by a mechanism that involved impairing the capacity of dendritic cells to instigate a T_H_2-cell-mediated allergic inflammation [89]. High fiber or acetate feeding was found to suppress allergic airway disease by HDAC inhibition and increased FOXP3 acetylation in adult mice, and this effect was conferrable to fetal mice, in which a high-fiber or acetate maternal diet was able to suppress the expression of certain genes related to asthma [90]. The Canadian Healthy Infant Longitudinal Development study found that infants at risk for asthma showed transient alterations in the composition of their gut microbiota compared to low-risk infants during the first 100 days of life [92]. These at-risk infants had reduced levels of microbial taxa involved in SCFA formation (specifically *Lachnospira*, *Veillonella*, *Faecalibacterium* and *Rothia*) and reduced fecal acetate. Inoculating germ-free mice with these four microbial taxa ameliorated airway inflammation in their offspring, demonstrating a causal role in suppressing inflammation [92]. Together, these results suggest the potential for introducing bacteria (or combinations of bacteria) that increase SCFA production as a measure to prevent the development of asthma and other related inflammatory diseases in both adults and children. Realization of this potential will require optimization of appropriate microbiota for supplementation and subsequent clinical trials.

### Effects on regulation of appetite and energy homeostasis

The landmark study by Turnbaugh and colleagues showing that transfer of microbiota from obese mice into germ-free mice increased adiposity and cecal levels of SCFAs relative to the transfer of microbiota from lean mice [12] has led to intense interest in the role of the microbial production of SCFAs in the regulation of appetite and energy homeostasis. Turnbaugh and colleagues attributed the obesegenic potential of transferred microbiota to its increased capacity to ferment dietary components to SCFAs, resulting in increased energy harvest. Subsequent studies showed obese humans have higher fecal SCFA levels than lean individuals [93] and that that roux-en-Y gastric bypass surgery, a highly effective treatment for obesity and type 2 diabetes, causes a significant change in fecal microbial profiles in humans and rodents and results in reduced levels of SCFAs [94–97]. While these results suggest that elevated microbial production of SCFAs promote obesity, a number of studies support an opposite conclusion. For instance, microbiota transfer experiments into germ-free mice from human twin donors where one was obese and one lean showed that, as with obese mice, phenotypes transferred with the microbiota; however, in this case transfer from lean donors resulted in higher cecal propionate and butyrate levels. Furthermore, diets enriched in inulin or other non-digestible fibers that increase formation of SCFAs consistently inhibit obesity in humans [98, 99]. Finally, direct administration of SCFAs, particularly butyrate, inhibits weight gain, adiposity, and insulin resistance in mice fed a high-fat diet (HFD) [41, 100–103].

A recent meta-analysis of various studies in this field by Byrne and colleagues led them to conclude that increasing SCFA levels had an overall net benefit on obesity due to their effects on satiety and reduced food intake, increased energy expenditure and thermogenesis, and inhibition of lipogenesis and cholesterol synthesis [104]. One molecular mechanism underlying the anti-obesity effect of SCFAs is improved barrier function, which prevents the passage of bacterial toxins into the circulation, inducing metabolic endotoxemia, obesity, and insulin resistance [105–107]. Additionally, SCFA activation of AMPK and GPR43 induces multiple responses that can reduce adiposity, including increasing fatty oxidation, decreasing glucose levels, and increasing secretion of satiety-inducing peptides such as GLP-1, peptide YY (PYY), and gastric inhibitory polypeptide (GIP) (reviewed in [34]).

Since, on the whole, increasing the microbial production of SCFAs appears to be a reasonable therapeutic intervention for the treatment of obesity, future studies are needed to determine how to effectively carry out such long-term interventions in humans. For a significant fraction of the human population, consumption of large amounts of non-digestible dietary fibers such as inulin is significantly hampered by undesirable gastrointestinal effects such as bloating, abdominal cramping, flatulence and diarrhea. These adverse effects result in part from the simultaneous formation of hydrogen gas and carbon dioxide during fermentation. Therefore, optimization of the microbiota or substrates to enhance SCFA production and to minimize released hydrogen and carbon dioxide gas will be critical for the wide-spread application of this treatment in the general population.

## Trimethylamine and trimethylamine *N*-oxide

TMA and its co-metabolite TMAO were identified by screening metabolites associated with cardiovascular disease (CVD), and TMA was shown to require gut bacteria for its formation [15]. Cleavage of choline to TMA and acetaldehyde by two enzymes originally identified in *Desulfovibrio desulfuricans*, CutC and CutD, allow choline to be used as an energy source [108]. Recent studies found homologous genes in a variety of *Proteobacteria* and *Firmicutes*, and to a much lesser extent *Actinobacteria*, suggesting spread via horizontal gene transfer [109]. TMA was also recently shown to form from l-carnitine and choline via an analogous reaction catalyzed by the YeaW and YeaX enzymes originally characterized in *Escherichia coli* [110], and by CntA and CntB, originally characterized in *Acinetobacter baumannii* [111]. After formation and absorption in the colon, TMA passes into the portal circulation, which directs blood into the liver, where it is oxidized to TMAO by flavin-containing mono-oxygenase 3 (FMO3) [112]. Analysis of genetic variation among inbred strains of mice indicates that plasma TMAO levels significantly correlate with FMO3 activity [112]. Oral antibiotics block the increase in TMAO that normally occurs after dietary challenge with either choline or carnitine, demonstrating that the generation of TMAO requires microbial bacteria [15, 113, 114].

TMAO levels predict risk for atherosclerosis [15, 112, 115], and are elevated in patients with chronic kidney disease (CKD) [116] and obesity [17, 98], and decreased in ulcerative colitis [117]. TMAO directly induces CVD, as administration of TMAO itself or of sufficient choline or l-carnitine to raise TMAO levels can all increase atherosclerosis in *Apoe*^*−/−*^ mice [15, 114]. The specific molecular mechanisms by which TMAO exerts its pathological effects are currently unknown. Accumulation of TMAO in the kidney may alter osmotic balance and elevated TMAO levels associate in animal models with markers of renal damage such fibrosis and dysfunction [116]. Thus far, no receptor for TMAO has been identified. TMA, but not TMAO, acts as a ligand for trace amine-associated receptor 5 (TAAR5) [118], but TAAR5 appears to be exclusively expressed in the olfactory sensory neurons. Administration of TMAO to *Apoe*^*−*/*−*^ mice inhibits reverse cholesterol transport from macrophages in vivo [114], but treating macrophages directly with TMAO in cell culture does not increase their ability to take up cholesterol or inhibit their ability to efflux cholesterol to ApoA1 or HDL [119]. Reduction of FMO3 activity (which increases TMA levels and decreases TMAO levels) decreases intestinal cholesterol absorption, reduces hepatic biliary secretion and LXR signaling, and increases cholesterol disposal via transintestinal cholesterol efflux (active secretion of cholesterol from the small intestine) [120]. Administering antibiotics blocks these effects, while TMAO supplementation does not, suggesting that the effects of reducing FMO3 activity resulted from increased TMA or another microbial substrate of FMO3 [120]. Thus, studies elucidating the molecular targets of TMAO and the potential roles of TMA are greatly needed.

Without identified TMAO molecular targets, interventions to reduce CVD must focus on reducing TMAO levels. Reducing dietary choline or l-carnitine would lower TMAO levels, but may have undesirable effects. In particular, supplementation with lower levels of l-carnitine than needed for TMAO formation may improve cardiovascular function [121]. A meta-analysis of 13 controlled trials (*n* = 3629) showed that l-carnitine supplementation reduces all-cause mortality by 27 % [122]. While potentially beneficial for cardiovascular health, choline deficiency markedly increases risk for non-alcoholic liver disease. Inhibiting FMO3 to reduce TMAO levels is also undesirable, as accumulation of TMA results in fish malodor disorder. Because of these limitations, current pharmaceutical development is focusing on a revolutionary approach: non-lethal targeting of microbes by selectively inhibiting pathways detrimental to their host, such as microbial CutC/D, CntA/B and YeaW/X. A structural analog of choline, 3,3-dimethyl-1-butanol (DMB), non-lethally inhibits microbial CutC/D and reduces TMAO levels in mice fed a high-choline or l-carnitine diet [123]. Importantly, DMB inhibits macrophage foam cell formation and atherosclerotic lesion development in *Apoe*^*−/−*^ mice [123]. Future clinical trials are needed to determine the safety and efficacy of CutC/D inhibitors in reducing TMAO levels and disease in humans, as well as whether resistance to their effects will occur with long-term treatment strategies. Nevertheless, this revolutionary strategy of selective, non-lethal inhibition of microbial function likely represents an important new front in the pharmacological treatment of human diseases.

## Tryptophan metabolites: indole and indole derivatives

**Fig. 1:**
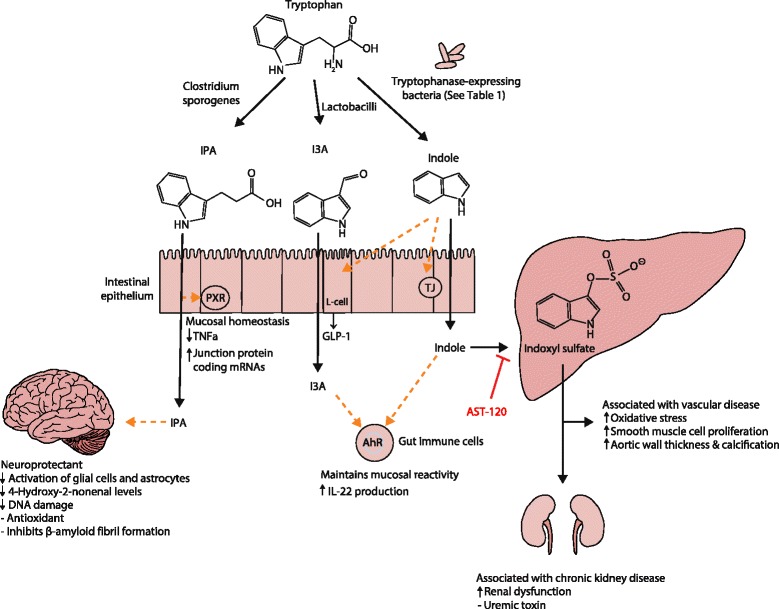
Molecular mechanisms of action of indole and its metabolites on host physiology and disease. Tryptophan in the colonic lumen is catabolized by bacteria to yield indole and indole derivatives. Indole-3-propionate (*IPA*) acts on intestinal cells via pregnane X receptors (*PXR*) to maintain mucosal homeostasis and barrier function. IPA can also act on other organs such as the brain, where it confers neuroprotective effects against ischemia-induced neuronal damage or against Alzheimer’s disease. Indole-3-aldehyde (*I3A*) acts on the aryl hydrocarbon receptor (*AhR*) found on intestinal immune cells and increases interleukin-22 (*IL-22*) production. Activation of AhR plays a crucial role in gut immunity, such as in maintaining the epithelial barrier function and promoting immune tolerance to promote microbial commensalism while protecting against pathogenic infections. Indole has a number of roles, such as a signaling molecule to intestinal L cells to produce glucagon-like protein 1 (*GLP-1*) or as a ligand for AhR. Indole is also metabolized by the liver to indoxyl sulfate, where an excess is detrimental to human health. Accumulation of indoxyl sulfate in physiologic fluid is toxic and associated with vascular disease and renal dysfunction. AST-120, an orally administered intestinal sorbent, adsorbs indole and decreases serum concentrations of indoxyl sulfate, and is a potential treatment for managing chronic kidney disease

Tryptophan is an essential amino acid found in a variety of foods such as red meat, fish and eggs. Commensal bacteria expressing tryptophanase catabolize tryptophan to indole, a quorum-sensing compound for bacteria [124] (Fig. 1). *Lactobacillus* spp. convert tryptophan to indole-3-aldehyde (I3A) through unidentified enzymes [125]. *Clostridium sporogenes* convert tryptophan to IPA [6], likely via a tryptophan deaminase. After absorption from the intestinal tract into portal circulation, the liver converts indole to IndS.

Indole and its metabolites affect host physiology via a number of molecular mechanisms (Fig. 1). Indole and I3A are agonists for the aryl hydrocarbon receptor (AhR), a transcription factor that regulates interleukin (IL)-22 expression, increases T_H_17-cell activity, and helps maintain intraepithelial lymphocytes [125]. Indole upregulates the expression of tight junction proteins and modulates the expressions of pro- and anti-inflammatory genes in intestinal epithelial cells [126, 127]. These activities of AhR help ensure that commensal bacteria outcompete pathogenic bacteria in the gut microbiota [128], and the absence of AhR increases the severity of DSS-induced colitis [129] and response to *Citrobacter rodentium* infection [130] (a model of human enteropathogenic *E. coli* infections). In addition to these effects, recent studies show that indole also modulates GLP-1 release from L cells [131], so that indole formation may contribute to satiety and inhibition of obesity. Other recent studies demonstrate that IPA is a pregnane X receptor (PXR) agonist, particularly in the presence of indole [132]. A wide-range of PXR agonists inhibit NF-κB [133], and downregulation of intestinal tumor necrosis factor (TNF)-α and upregulation of junction proteins by IPA requires PXR [132]. IPA also potently scavenges hydroxyl radicals [134], thereby protecting against oxidative injury in various animal models [134–137]. Thus, future studies are needed to determine if enhancing IPA formation by bacteria or directly administering IPA is beneficial in inflammatory conditions such as inflammatory bowel disease and colorectal cancer.

While indole appears to be primarily beneficial, its metabolite IndS is a uremic toxin that accumulates in patients with CKD [138]. IndS is also associated with accelerated glomerular sclerosis [139], enhanced endothelial dysfunction [140], enhanced monocyte adhesion to the vascular endothelium [141], and increased oxidative stress [141, 142]. The oral charcoal adsorbent AST-120 binds indoles in the gut lumen and reduces plasma IndS levels, thereby reducing kidney damage and atherosclerosis associated with kidney injury [143]. Future studies are needed to determine if diverting tryptophan metabolism away from IndS towards IPA will be beneficial in renal disease or other conditions.

## Tyrosine metabolites: para-cresyl sulfate and 4-ethylphenylsulfate

PCS and 4-ethylphenyl sulfate (EPS) are structurally similar uremic toxins formed by hepatic sulfation of the microbial metabolites para-cresol and 4-ethylphenol, respectively. The lack of PCS or EPS in the plasma and urine of germ-free mice demonstrates their microbial origins. Inactivating mutants of the hydroxyphenylacetate decarboxylase operon genes (*hpdB/C/A*) from *Clostridium difficile* prevent fermentation of tyrosine or its metabolite hydroxyphenylacetate to para-cresol [144]. Few other gut bacteria encode HpdB/C/A [144]. Bacterial pathways for formation of 4-ethylphenol have not yet been characterized, but the wine spoilage yeast *Brettanomyces* generates 4-ethylphenol from the tyrosine metabolite para-coumaric acid that is present in many foods via cinnamate decarboxylase and vinyl phenol reductase. 4-Ethylphenol also forms from orally administered genistein, a phytoestrogen found in soy, by uncharacterized but presumably microbial pathways [145].

Both PCS and EPS accumulate in patients with severe CKD undergoing hemodialysis [146]. PCS levels predict clinical outcomes in patients with CKD [147] and correlate with cardiovascular mortality in CKD patients [148, 149]. While conventional dialysis fails to remove PCS, treatment with the oral adsorbent AST-120 [150] or with the prebiotic arabino-xylo-oligosaccharide [151] lowers plasma PCS levels. Vegetarians have lower levels of PCS than omnivores [152]. There are very few studies of EPS. EPS levels are elevated in a rat model of chronic renal failure and AST-120 treatment lowers these levels [153]. EPS levels increase 46-fold in a mouse model of autism and treatment with *Bacteroides fragilis* blocks this increase [28]. Administration of EPS to mice results in anxiety-like behaviors [28].

Molecular mechanisms of action ascribed to PCS include direct damage of cell membranes [154], induction of apoptotic pathways [155], activation of NADPH oxidase 4 (NOX4) resulting in reactive oxygen species (ROS) formation [156], activation of JNK and p38-MAPK [157], activation of Rho-kinase (ROCK) leading to endothelial damage [158], activation of epidermal growth factor (EGF) receptor leading to expression of matrix metalloproteinases 2 and 9 [159], and inhibition of a variety of drug-metabolizing enzymes including CYP2E1, CYP3A4, UGT1A1, UGT1A9, and UGT2B7 [160]. Given its chemical similarity to PCS, EPS is expected to exert similar effects, but no specific molecular targets have been demonstrated to date. Future studies are needed to identify pharmaceutical inhibitors of the PCS and EPS biosynthetic pathways and whether such inhibitors have beneficial effects in disease.

## Essential fatty acid-derived metabolites

The microbiota of ruminants have long been known to transform the essential fatty acids linoleic acid (LA) and linolenic acid to CLAs such as *cis*-9 and *trans*-11 CLA, and conjugate linolenic acids (CLnAs) such as *cis*-9, *trans*-11 and *cis*-15 CLnA, respectively [161–163], via the action of isomerases. However, recent studies found that the microbiota of mice and humans, particularly *Lachnospiraceae*, *Lactobacillus* spp. and *Bifidobacteria*, possess the capacity to generate both CLAs and CLnAs [164–166]. In *Lactobacillus*, intermediates for the formation of conjugated fatty acids include the oxygenated metabolites HYA and 10-hydroxyoctadecanoate (HYB) [167, 168]. The enzymes involved in the transformation of LA to CLAs by *Lactobacillus* were recently characterized and include myosin-cross-reactive antigen, short-chain dehydrogenase/oxidoreductase, and acetoacetate decarboxylase [169].

Conjugated fatty acids exert many highly beneficial effects, including reduction of adiposity, improved insulin sensitivity, reduced carcinogenesis, and reduced atherosclerosis (reviewed in [170]). CLAs and CLnAs act via PPAR-γ (reviewed in [171]), PPAR-α [172], and inhibition of cyclooxygenases and lipoxygenases [173, 174]. Whether typical intestinal microbiota generate sufficient CLA/CLnA to exert the extraintestinal effects seen with CLA/CLnA supplementation is unclear, as feeding essential fatty acids increases gut but not circulating levels of CLAs and CLnAs [164]. Like CLAs and CLnAs, HYA also exerts anti-inflammatory activities, including downregulating lipopolysaccharide (LPS)-induced maturation of dendritic cells, blocking TNF-induced barrier impairment, and protecting against DSS-induced intestinal injury [175, 176]. HYA acts via the GPR40–MEK–ERK pathway [176]. Future studies are needed to determine if increasing microbial HYA production can be used therapeutically.

## Translation to future diagnostics and therapeutics

In previous sections, we have touched briefly on potential future studies for individual metabolites, but there are additional developments needed in broad areas of research and understanding to fully realize the potential of gut microbial metabolites for disease treatment. We will conclude by highlighting four of these needed developments.

First, the development of minimal sets of biomarker microbial metabolites that identify particular disease states or that distinguish between closely related disease conditions. The analysis carried out by de Preter and colleagues for inflammatory bowel disease is proof of principal for this strategy [22], and similar approaches for highly heterogeneous conditions such as autism spectrum disorder, in which the microbiota has also been implicated [177], might be even more valuable. This also applies to the identification of individuals who might be at risk for disease, such as was found for individuals who carried high levels of bacterial strains that converted cholesterol to coprostanol that made them more vulnerable to *C. difficile* infections. For translation to actual treatment, measurements will need to be carried out in clinical laboratories in which immunoassay arrays, rather than the more sophisticated MS or NMR methods available in research settings, will likely continue to be the primary methods available. Thus, identifying the minimal number of biomarker metabolites needed to selectively assess a condition is critical. Similar strategies can be used to determine the efficacy and safety of interventions.

Second, the development of algorithms to predict personalized responses to dietary and pharmaceutical interventions based on microbial metabolites. An exciting example of this approach was recently reported by Zeevi and colleagues, who demonstrated that the highly variable glycemic response of different individuals to the same foods could be predicted using their gut microbiota and other data [32]. Similarly, being able to predict the responses of specific metabolites such as SCFAs to individual foods using tools such as CASINO [31] may be critical for allowing individuals with intolerance for particular dietary components to successfully use functional foods to increase colonic levels of SCFAs. Algorithm-based personalization seems essential for any nutrition-based approaches, given the variability of microbial composition among individuals.

Third, the development of readily generalizable methods to increase gut microbial production of beneficial metabolites, either by selectively increasing the abundance of native species that produce that metabolite or by engineering endogenous gut microbiota to produce it in high levels. An example of this latter approach is our study using heterologous expression of the satiety factor *N*-acylphosphatidylethanolamine in commensal *E. coli* (strain Nissle 1917), leading to inhibition of obesity in mice fed a HFD [178]. Such strategies might be helpful to produce sufficient IPA, CLA or HYA to block inflammatory diseases, but could also be utilized to test novel metabolites as they are identified. One advantage of engineered bacteria may be the ability to produce beneficial metabolites in bacterial strains that colonize well in the gut of a diseased individual in the place of native bacteria that produce these same beneficial metabolites but poorly colonize in the diseased gut.

Fourth, the development of non-lethal specific inhibitors for various microbial pathways that produce harmful metabolites, similar to work done with CutC/D. In particular, inhibition of the formation of para-creysl and 4-ethylphenol appear amendable to this strategy. This revolutionary approach to controlling harmful bacterial metabolites seems unlikely to result in the rapid evolution of resistance that occurs with standard antibiotics, since there is a much more limited fitness advantage of carrying resistance. If this is the case, then long-term use of such metabolic pathway inhibitors will have great potential benefit in chronic diseases.

## Conclusions and future perspectives

The past decade has seen remarkable progress in our understanding of the significant role that gut microbial metabolites play in modulating the health of their hosts. MS and NMR studies have identified a significant number of microbial metabolites that differ in disease conditions, and these same methods are now being exploited to better identify subtle differences in closely related diseases. Some of these identified metabolites, such as TMAO, IndS and PCS, appear to directly increase susceptibility to disease, while others, such as SCFA, IPA, CLA and HYA, appear to exert protective effects. Much work remains to fully characterize the physiological effects of these and the many other microbial metabolites that may be important in human health. It seems highly likely that future studies will identify many other disease states in which gut microbial metabolites are significantly enriched or depleted. It is important to keep in mind that by themselves such studies do not demonstrate causality. Thus, it seems there is a considerable need for carefully controlled studies to determine the physiological effects of each identified microbial metabolite and its specific mechanisms of action. Furthermore, in order to fully exploit the potential of the gut microbiota for disease prevention, we need a much greater understanding of how dietary components and host genetics affect the production of various metabolites. Finally, translation of these findings to clinical practice will require the development of widely available clinical chemistry methods to detect changes in an individual’s key metabolites. Despite these tremendous challenges to fully exploiting the gut microbiota for human health, the remarkable progress of the last decade suggests that such approaches have significant potential to revolutionize therapeutic approaches to human disease.

## Abbreviations

AhR: aryl hydrocarbon receptor
AMPK: AMP kinase
CKD: chronic kidney disease
CLA: conjugated linoleic acid
CLnA: conjugated linolenic acid
CoA: coenzyme A
CVD: cardiovascular disease
DMB: 3,3-dimethyl-1-butanol
DSS: dextran sodium sulfate
EPS: 4-ethylphenylsulfate
GIP: gastric inhibitory polypeptide
GLP: glucagon-like peptide
GPR: G-protein-coupled receptor
HDAC: histone deacetylase
HFD: high-fat diet
HYA: 10-hydroxy-*cis*-12-octadecenoate
HYB: 10-hydroxyoctadecanoate
I3A: indole-3-aldehyde
IL: interleukin
IndS: indoxyl sulfate
IPA: indole-3-propionate
LA: linoleic acid
LPS: lipopolysaccharide
MS: mass spectrometry
NMR: nuclear magnetic resonance
PCS: para-cresyl sulfate
PPAR-γ: peroxisome proliferator-activated receptor-γ
PYY: Peptide YY
PXR: pregnane X receptor
ROCK: Rho-kinase
ROS: reactive oxygen species
SCFA: short-chain fatty acid
TMA: trimethylamine
TMAO: trimethylamine *N*-oxide
TNF: tumor necrosis factor
